# The Short-Term Variation of Human Gut Mycobiome in Response to Dietary Intervention of Different Macronutrient Distributions

**DOI:** 10.3390/nu15092152

**Published:** 2023-04-29

**Authors:** Yunyi Tian, Wanglong Gou, Yue Ma, Menglei Shuai, Xinxiu Liang, Yuanqing Fu, Ju-Sheng Zheng

**Affiliations:** 1 School of Medicine, Zhejiang University, Hangzhou 310058, China; tianyunyi@westlake.edu.cn; 2 Research Center for Industries of the Future, Key Laboratory of Growth Regulation and Translational Research of Zhejiang Province, School of Life Sciences, Westlake University, Hangzhou 310030, China; 3 Westlake Intelligent Biomarker Discovery Lab, Westlake Laboratory of Life Sciences and Biomedicine, Hangzhou 310030, China; 4 Institute of Basic Medical Sciences, Westlake Institute for Advanced Study, Hangzhou 310030, China

**Keywords:** gut mycobiome, high-carbohydrate diet, low-carbohydrate diet, macronutrients

## Abstract

While the human gut is home to a complex and diverse community of microbes, including bacteria and fungi, research on the gut microbiome has largely focused on bacteria, with relatively little attention given to the gut mycobiome. This study aims to investigate how diets with different dietary macronutrient distributions impact the gut mycobiome. We investigated gut mycobiome response to high-carbohydrate, low-fat (HC) and low-carbohydrate high-fat (LC) diet interventions based on a series of 72-day feeding-based n-of-1 clinical trials. A total of 30 participants were enrolled and underwent three sets of HC and LC dietary interventions in a randomized sequence. Each set lasted for 24 days with a 6-day washout period between dietary interventions. We collected and analyzed the fungal composition of 317 stool samples before and after each intervention period. To account for intra-individual variation across the three sets, we averaged the mycobiome data from the repeated sets for analysis. Of the 30 participants, 28 (aged 22–34 years) completed the entire intervention. Our results revealed a significant increase in gut fungal alpha diversity (*p* < 0.05) and significant changes in fungal composition (beta diversity, *p* < 0.05) after the HC dietary intervention. Specifically, we observed the enrichment of five fungal genera (*Pleurotus*, *Kazachstania*, *Auricularia*, *Paraphaeosphaeria*, *Ustilaginaceae* sp.; FDR < 0.052) and depletion of one fungal genus (*Blumeria*; FDR = 0.03) after the HC intervention. After the LC dietary intervention, one fungal genus was enriched (*Ustilaginaceae* sp.; FDR = 0.003), and five fungal genera were depleted (*Blumeria, Agaricomycetes* spp., *Malassezia*, *Rhizopus*, and *Penicillium*; FDR < 0.1). This study provides novel evidence on how the gut mycobiome structure and composition change in response to the HC and LC dietary interventions and reveals diet-specific changes in the fungal genera.

## 1. Introduction

The human gut is inhabited by a diverse and complex community of microbes, such as bacteria, viruses, and fungi, which are critical in maintaining human health. These microorganisms play a vital role in intestinal barrier function, host neurological function, immune system modulation and physiological processes, especially in regulating blood glucose [1,2,3,4]. While previous studies focus on commensal bacteria, recent research reveals the importance of the gut mycobiota (i.e., fungal microbiota) in human health and diseases. Gut mycobiome is less diverse and abundant than bacteria in the gut, which comprises approximately 0.1% of the total gut microbes and inhabits symbiotically with bacteria as a commensal in the human gut [5]. Studies have indicated that dysbiosis of gut mycobiota may contribute to several complex diseases, including inflammatory bowel disease, enterocolitis and colorectal cancers, and diabetes [6,7,8].

Like the bacterial microbiome, the gut mycobiome is shaped by various environmental factors, including diet [9,10]. Studies have shown that the types and quantities of dietary nutrients may have an impact on the composition and diversity of both bacterial and fungal communities in the gut [11,12]. Nevertheless, the influence of diet on the gut mycobiome is still not well understood. The availability of carbohydrates in the diet can directly influence the growth and proliferation of certain fungal species in the gut. Recent consumption of carbohydrate-rich food by healthy humans has been correlated with an increase in *Candida* spp. Colonization influences the proportion of commensal opportunistic pathogens in the gut [13]. The composition and long-term stability of gut mycobiome have been investigated in prior studies. Human cohort studies have reported inconsistent associations of dietary factors with gut mycobiome, and the results are mostly descriptive, with little causal evidence [14,15]. Therefore, more research is needed to understand the effects of diet on the gut mycobiome and how this relationship impacts human health. In particular, feeding interventional studies with different macronutrient distributions (all foods are provided to the participants) may have a unique role in demonstrating the influence of diet on gut mycobiome, yet this sort of intervention is rare.

In the present study, we aimed to explore short-term gut mycobiome variations in response to a high-carbohydrate, low-fat (HC) diet and a low-carbohydrate, high-fat (LC) diet in a cross-over N-of-1 feeding trial among 30 participants over 72 days. The required macronutrient distributions were achieved mainly by modifying the grain consumption, including wheat flour and white rice. As an exploratory analysis, we explored the association of the diet-related gut fungi with dynamics of glycemic traits measured by continuous glucose monitoring during the interventions.

## 2. Materials and Methods

### 2.1. Study Design and Participants

The Westlake n-of-1 Trials for Macronutrient Intake study (WE-MACNUTR) investigated postprandial glycemic as well as human gut microbial responses to the interventional diets with different macronutrients distributions (HC vs. LC), which had been reported previously [16]. Briefly, the WE-MACNUTR study enrolled 30 adults from the Westlake University at Hangzhou in south China between October 2019 and December 2019, of which 28 participants completed the dietary intervention and were included in the present analysis. This trial consisted of three cycles, and each cycle included a 6-day HC dietary intervention and a 6-day LC dietary intervention with a 6-day washout period following each intervention. The diets were isocaloric, with carbohydrate and fat contents as their primary distinguishing features. The HC diet consisted of 65–75% of total energy intake (E) from carbohydrates, while the LC diet consisted of 15–25% E from carbohydrates. In all dietary interventions, 15% of the energy intake was from protein. The required macronutrient distributions were achieved mainly by modifying the intake of grain consumption, including wheat flour and rice.

**Figure 1:**
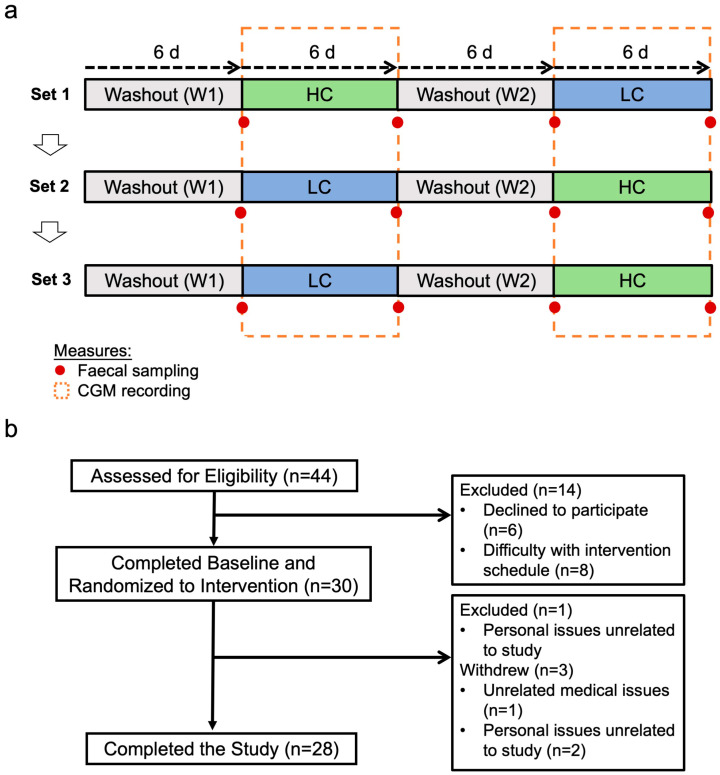
Study design and flow-chart. (**a**) Study design schematic of clinical trial; (**b**) CONSORT diagram describing the number of participants throughout the study. An HC and an LC diet were completed in a randomized order separated by a 6-day washout period. Participants (n = 28) visited the study site on 12 occasions along with fecal samplings. Continuous glucose monitors were worn by participants throughout the 6-day intervention periods (36 days in total).

At the beginning of the trial, eligibility of participants was screened based on anthropometric measurements and lifestyle questionnaires, including habitual diet, physical activity, mood, sleep patterns, and medical history at baseline visit. Participants wore digital devices to measure interstitial glucose via continuous glucose monitor (CGM; Freestyle Libre, Abbott, Abbott Park, IL, USA) two days before each dietary intervention and for the entire intervention period (six days). Five glycemic metrics were analyzed to examine different traits of the pre- and postprandial glycemic homeostasis: morning mean blood glucose (morning MBG, calculated from the CGM mean glucose values between 06:00 and 07:00 a.m.), the maximum postprandial glucose (MPG), the total area under the curve from 00:00 to 24:00 of the day, (AUC_24_), percentage of time spent in a hyperglycemic range (PTH, clinically set glucose level > 10 mmol/L), and mean amplitude of glucose excursions (MAGE). Fecal samples were collected at all 12 visits to the study site. A summary of the study profile is shown in Figure 1, and detailed randomization and allocation have been extensively described elsewhere [17].

### 2.2. DNA Extraction and Sequencing

Fecal samples were collected by spatula into 60 mL sterile fecal collection containers and placed in the laboratory freezer (−20 °C) within 15 min of production. Thereafter, samples were homogenized, aliquoted and stored at −80 °C in 1.5 mL tubes (Qiagen, Germany) for later analysis of Internal Transcribed Spacer 2 (ITS2) sequencing [18]. The fungal DNA was first isolated from 200 mg of fecal samples using the E.Z.N.A.^®^ soil DNA Kit (Omega Bio-tek, Norcross, GA, USA). The fecal samples were first homogenized and lysed, and the isolations were conducted in triplicate following the manufacturer’s protocols. Purified DNA was eluted in an elution buffer and quantitated using NanoDrop 2000 UV-vis spectrophotometer (Thermo Scientific, Wilmington, DE, USA). The quality of the purified DNA was analyzed by 1% agarose gel electrophoresis.

The fungal ITS rRNA gene was amplified by performing real-time PCR (ABI GeneAmp^®^ 9700, Foster City, CA, USA) with primers ITS3F and ITS4R on appropriate dilutions of the purified DNA [19]. Briefly, the PCR amplification reactions were as follows: initial denaturation at 95 °C for 3 min, 27 amplification cycles of denaturing at 95 °C for 30 s, annealing at 55 °C for 30 s and extension at 72 °C for 45 s, followed by a final extension at 72 °C for 10 min. The resulting PCR products were then extracted from a 2% agarose gel electrophoresis, purified using the AxyPrep DNA Gel Extraction Kit (Axygen Biosciences, Union City, CA, USA), and quantified using QuantiFluor™-ST (Promega, Madison, WI, USA). Samples were then sequenced on the MiSeq platform (Illumina Inc., San Diego, CA, USA) using the paired-end sequenced kit (600 cycles) [20].

### 2.3. Bioinformatic Analysis

Raw sequence reads were demultiplexed and further processed with QIIME2 (version 2020.2) [21]. After denoising and chimera removal, the demultiplexed ITS2 sequences were grouped into amplicon sequence variants (ASVs) using DADA2 [22]. The ASV features present in only one sample were excluded. The individual ASVs were taxonomically classified based on the UNITE (version 8.2, 99%) database using the VSEARCH tool wrapped in QIIME2.

The dataset was rarefied at a sampling depth of 4500 before computing diversity metrics to avoid bias. Alpha diversity indices were estimated by the Shannon index (a quantitative measure of community diversity), observed features (a qualitative measure of community richness), and Gini-Simpson (a measure of community evenness). Beta diversity was calculated using Bray–Curtis dissimilarity index. Analysis and visualization of fungal communities were conducted in R (version 4.1.0).

### 2.4. Statistical Analysis

All statistical analyses were performed using Stata (version 15.0) or R (version 4.1.0). The baseline characteristics, including baseline demographic factors and fasting blood parameters of the study participants (n = 28), were presented as mean (standard deviation) or number (%). We then depicted the gut mycobiome dynamics and profiles over the three intervention sets at both group and individual levels. Due to the n-of-1 design of the trial, we are also interested to see the gut mycobiome dynamics over time for each participant. At the individual level, we described the gut fungal genera detected among ≥50% of the sampling points of each participant. In the following statistical analysis, we only conducted a group-level comparison to achieve the aim of the present study (i.e., to investigate the overall effect of dietary macronutrient distributions on gut mycobiome).

Among the 28 participants who completed the trial, we compared the gut fungal alpha diversity before and after each intervention by paired Wilcoxon–Mann–Whitney test. The Bray–Curtis dissimilarity matrix was calculated based on the composition at the genus level using the *vegdist()* function from the *vegan* R package. Beta diversity was compared by permutational multivariate analysis of variance (PERMANOVA; *adonis()* function from *vegan* function; permutations = 999) based on the Bray–Curtis dissimilarities. As we repeated the same interventions across the three study sets, we took the average of the gut mycobiome across the three sets for the comparison analysis of fungal diversity before and after the diet interventions (HC or LC dietary intervention).

Next, we used the paired Wilcoxon–Mann–Whitney test to compare the mean differences in fungal genera before and after the dietary interventions (HC or LC dietary intervention). Gut fungal genera present in less than 5% of the stool samples were not considered for statistical analysis. All *p* values were corrected for false discovery rate (FDR) through the Benjamini–Hochberg procedure, and FDR < 0.1 was considered as potentially altered fungal genera after the intervention.

In our exploratory analysis, we examined the association of the above diet-related gut fungi with postprandial glycemic responses under different dietary interventions (either the HC or LC diet) by using linear mixed-effect models accounting for repeated measures (multiple time points per participant): dependent variable ~ (intercept) + independent variable + age + sex + BMI + (1| subject). Gut fungal genera were included as fixed effects, and the subject ID was adjusted as a random effect. CGM data were tested for normality using the Shapiro–Wilk test and log-transformed where applicable. We averaged the gut mycobiome compositions before and after the diet interventions as well as the postprandial glycemic features at the corresponding study set for the subsequent correlation analysis. All CGM metrics were standardized to allow comparison across metrics. The model included five CGM metrics obtained by mean values from three HC and three LC diets separately as the dependent variables (MBG, MPG, AUC_24_, PTH, and MAGE). Statistical significance was considered as *p* values < 0.05.

## 3. Results

### 3.1. Community Profiles of Gut Mycobiome over Study Period

In total, 28 participants were included in the final analysis. Table 1 summarizes the descriptive characteristics of the study participants. We identified five fungal phyla, 18 classes, 41 orders, 72 families, and 102 genera in our collected fecal samples. A detailed list of genera can be found in Supplementary Table S1.

**Figure 2:**
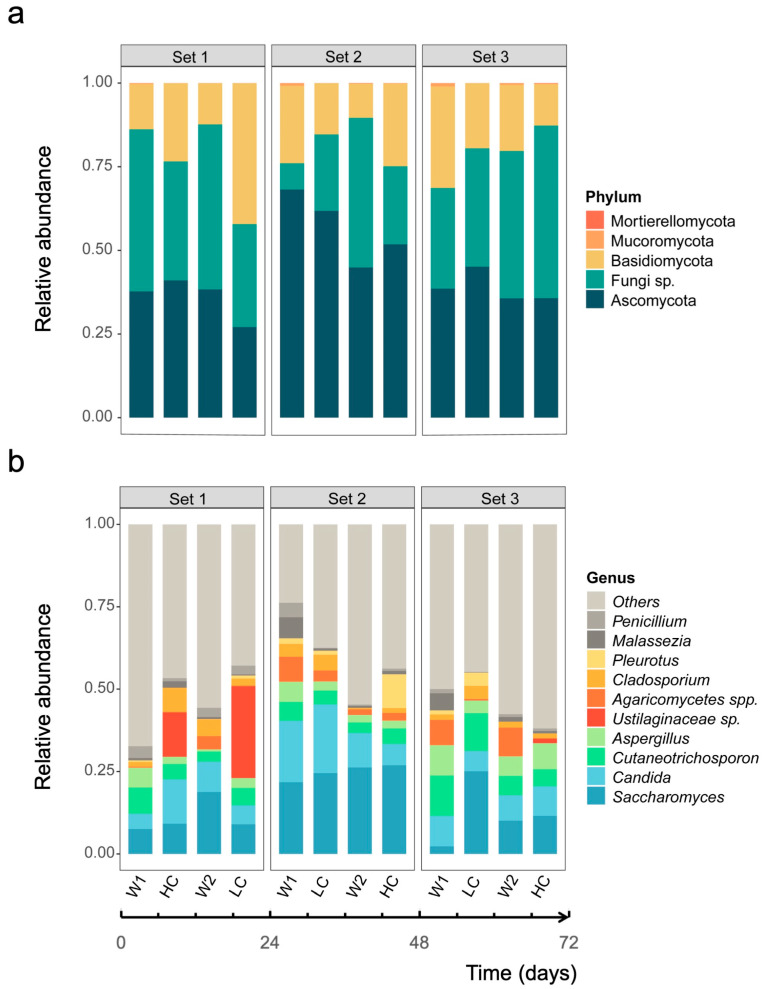
Community profiles of gut mycobiome during the interventions. (**a**) The relative abundance of gut fungal phyla; (**b**) The relative abundance of gut fungal genera. Only those of the top 10 genera are present here. The x-axis represents the trial arms grouped into three sets over the 72-day study period. “*Fungi* sp.” represents unidentified fungal phylum. “*Trichosporonaceae* sp.”, “*Agaricomycetes* spp.” and “*Ustilaginaceae* sp.” represent unidentified fungal genera. W1, the first washout; W2, the second washout.

The gut mycobiome composition primarily consisted of fungi from the *Ascomycota* and *Basidiomycota* phyla and unclassified Fungi sp., with a prevalence of 96.5%, 89.3% and 89.6%, respectively (Figure 2a, Supplementary Table S2). The relative abundance of the ten most abundant genera detected at each time point during the study is shown in Figure 2b. The identified fungal genera were mainly from *Ascomycota* and *Basidiomycota* phyla, with *Saccharomyces* being the most abundant genus among all samples, followed by *Candida*. Overall, among the top 10 abundant genera, five genera, including the two listed above as well as *Aspergillus*, *Cladosporium*, and *Penicillium*, belong to the *Ascomycota* phylum.

**Figure 3:**
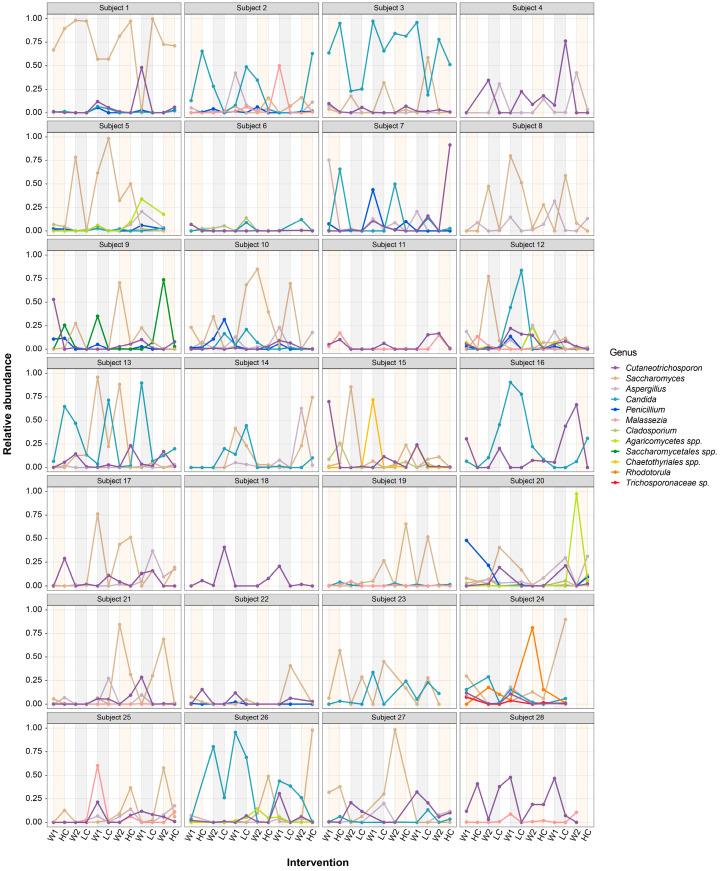
Descriptive overview of gut fungal genera for each individual over the 12 timepoints. In total, 10 to 12 fecal samples were analyzed per participant collected across the 12 timepoints, and only the fungal genera that were considered present were included in this plot. A fungal genus was considered present if detected among ≥ 50% of the sampling points. HC dietary interventions are shaded in light yellow, whereas LC dietary interventions are shaded in grey. “*Agaricomycetes* spp.”, “*Saccharomycetales* spp.”, “*Chaetothyriales* spp.”, and “*Trichosporonaceae* sp.” represent unidentified fungal genera. W1, the first washout; W2, the second washout.

Despite the participants following the same controlled diets, the relative abundance of fungal genera varied substantially across participants. We then depicted the gut fungal genera detected among ≥ 50% of the sampling points of each participant and observed that 12 genera fluctuated distinctly throughout the entire study, corresponding to the shift of intervention (Figure 3, Supplementary Table S3). The most abundant fungal genera were *Cutaneotrichosporon*, *Saccharomyces*, *Aspergillus* and *Candida*, which were detected in 22, 21, 17, and 16 participants, respectively. After 5% prevalence filtering, 31 core gut fungal genera were identified. The most prevalent genera were *Cutaneotrichosporon* (present in 59.4% of samples), followed by *Saccharomyces* (57.1%), *Aspergillus* (53.3%), *Candida* (52.4%), and *Malassezia* (37.5%). In addition, we identified *Cutaneotrichosporon*, *Saccharomyces*, *Aspergillus*, and *Malassezia* present at all sampling time points in all participants, and *Candida* was detected in 96.4% of participants (Supplementary Table S4). Overall, both interventions shared generally similar phylum profiles across the study period, and the top five fungal genera also showed a high degree of stability after dietary interventions.

### 3.2. Dynamic Changes in Human Gut Mycobiome Composition Induced by Dietary Intervention

**Figure 4:**
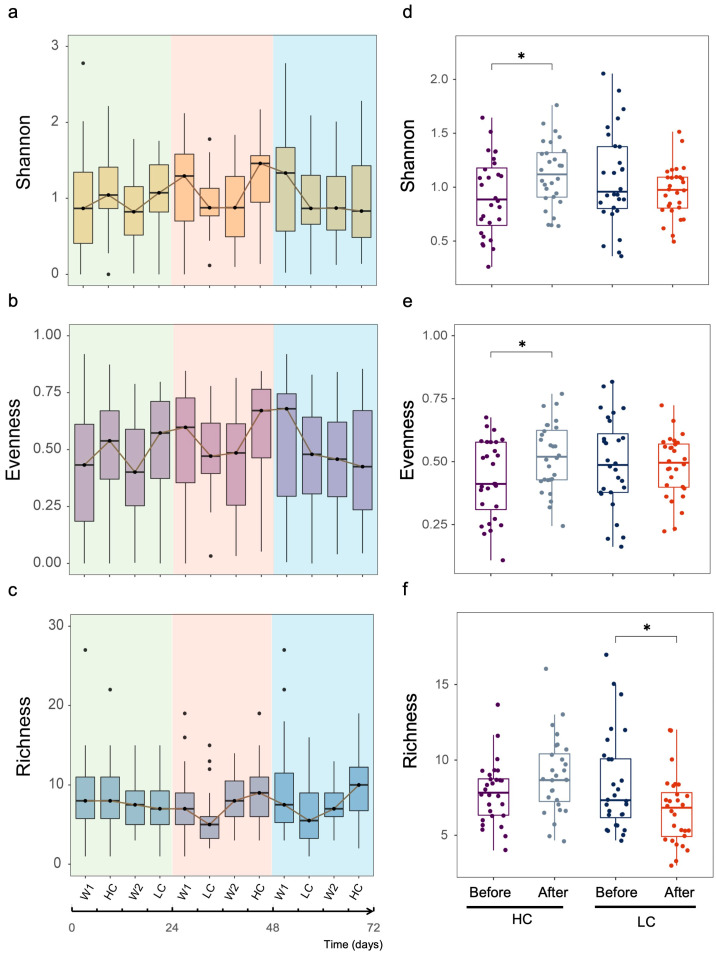
Changes of gut fungal alph -diversity during the dietary intervention. (**a**–**c**) are dynamics of the gut fungal alpha diversity during the whole study period. (**a**) Shannon index; (**b**) Evenness; (**c**) Richness (observed features). The study sets 1, 2, and 3 are shaded in green, pink, and blue, respectively. (**d**,**e**) Comparison of the gut fungal alpha diversity before and after the dietary interventions calculated from mean values of the three intervention sets. (**d**) Shannon index; (**e**) Evenness; (**f**) Richness. Each dot represents one participant. All box plots are expressed as the median with the interquartile range. All *p* values were calculated from Mann–Whitney test and the difference with statistical significance was denoted with an asterisk (*, *p* < 0.05). W1, the first washout; W2, the second washout.

Next, we investigated the fungal community variations in response to standardized diets over time. Fungal diversity (Shannon index) and richness (observed features) fluctuated remarkably in line with the shift between dietary interventions (Figure 4a–c). We observed a significantly higher diversity (Shannon index) and evenness after the HC diet (*p* = 0.034 and *p* = 0.036, respectively) as well as a decrease in richness after the LC diet (*p* = 0.019; Figure 4d,e).

**Figure 5:**
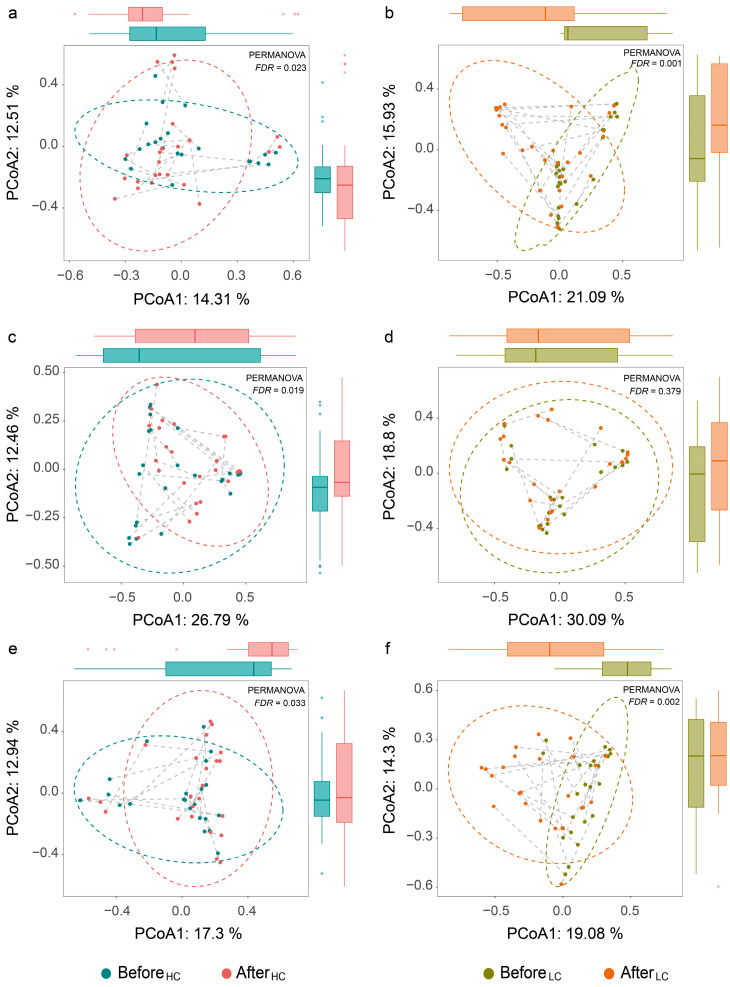
Principal coordinate analyses plot showing gut fungal community structure measured by Bray–Curtis dissimilarity before and after the dietary intervention. (**a**,**b**) in the study set 1; (**c**) and (**d**) in the study set 2; (**e**,**f**) in the study set 3. Each dot represents one participant. The dashed lines indicate the fecal samples originated from the same participant. The inter-individual distances (Bray–Curtis dissimilarity) were calculated between samples and were represented in principal coordinate analyses (PCoAs), with *p* values adjusted for multiple testing by using the Benjamini–Hochberg method for the false-discovery rate (FDR).

We measured variance in overall gut fungal composition in response to dietary interventions at each set. The principal coordinates analysis (PCoA) based on Bray–Curtis distances showed significant differences before and after the HC dietary interventions within each set (*p* < 0.05; Figure 5) as well as during the entire intervention period (*p* < 0.05; Supplementary Table S5).

### 3.3. Differential Effects between HC and LC Dietary Interventions on Gut Fungal Composition

**Figure 6:**
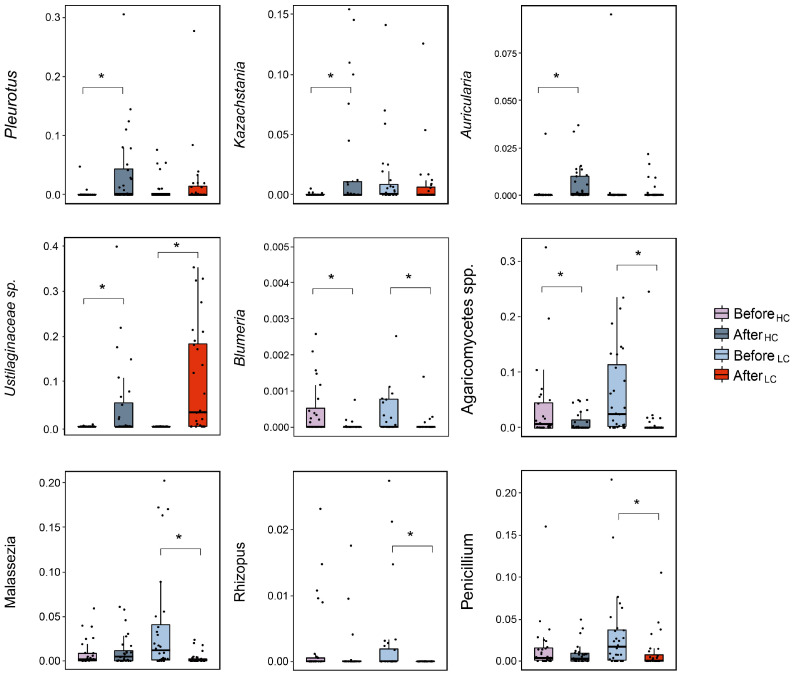
Changes in relative abundance of core diet-related fungi before and after the interventions. The box plots compare the relative abundance of 10 core diet-related fungi before and after the interventions calculated from mean values of the three intervention sets. All box plots are expressed as the median with the interquartile range. All *p* values were calculated from Mann–Whitney test, and the difference with statistical significance was denoted with an asterisk (*, *p* < 0.05).

We found four fungal genera enriched (*Pleurotus*, *Kazachstania*, *Auricularia*, *Ustilaginaceae* sp., FDR < 0.052) and one fungal genus depleted (*Blumeria*, FDR = 0.03) after HC dietary intervention. After the LC dietary intervention, one fungal genus was enriched (*Ustilaginaceae* sp., FDR = 0.003), and five fungal genera were depleted (*Blumeria, Agaricomycetes* spp.,* Malassezia*, *Rhizopus*, and *Penicillium*, FDR < 0.1) (Figure 6, Supplementary Tables S6 and S7). Among these fungal genera, *Blumeria* and *Agaricomycetes* spp. were decreased consistently in response to both dietary interventions. While the *Ustilaginaceae* sp. was increased consistently after both interventions.

### 3.4. Associations of Gut Fungi with Postprandial Glycemic Responses to Dietary Intervention

**Figure 7:**
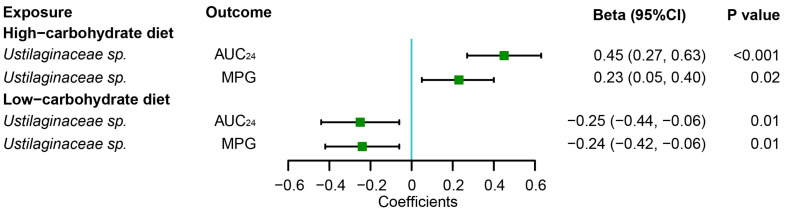
The association between the gut mycobiota and glycemic phenotypes by the dietary intervention period. Forest plot shows the interaction of dietary intervention with gut mycobiota on glycemic phenotypes. Fungal relative abundance and CGM features were calculated as mean values during each HC or LC dietary intervention separately. Associations were expressed as the difference in glycemic traits per 1 SD increment of gut mycobiome abundance. Linear mixed models were performed, adjusted for age, gender, and BMI. Abbreviations: MPG, maximum postprandial glucose; AUC_24_, the total area under the curve from 00:00 to 24:00 of the day.

We found 12 nominally significant associations between the above-identified gut fungi and glycemic metrics (*p* < 0.05, Supplementary Tables S8 and S9). Interestingly, the relative abundance of *Ustilaginaceae* sp. was positively associated with AUC_24_ and MPG (*p* < 0.001 and *p* = 0.016, respectively) on the HC diet but negatively associated with AUC_24_ and MPG on the LC diet (*p* = 0.012 and *p* = 0.010, respectively) (Figure 7).

## 4. Discussion

In a series of n-of-1 trials, we profiled the stabilities and dynamics of gut fungi in response to dietary interventions at both individual and population levels. The HC diet, but not the LC diet, may increase the fungal alpha diversity at the populational level. Both dietary interventions may affect the gut fungal community structure (i.e., beta diversity). We identified four gut fungal genera that were enriched after the HC diet and one genus enriched after the LC diet. We identified one fungal genus depleted after the HC diet and five genera depleted after the LC diet. Our results further suggest that the fungus *Ustilaginaceae* sp. was associated with glycemic traits in opposite directions depending on the dietary environment: a high-carbohydrate diet environment may stimulate the positive fungus-glycemic trait association, while a low-carbohydrate diet environment may have an opposite role. Our findings highlight the role of the dietary macronutrient distributions in shaping the diversity and composition of the fungal community and may even stimulate the health effect of specific fungi in the gut.

Advancements in microbial detection methods have improved our knowledge of gut mycobiome. According to current databases, the gut mycobiome can be detected in the lower gastrointestinal tract of around 70% of the population, and at least 267 fungal species have been identified in the intestines [23,24]. Previous research has shown that human gut mycobiome is mainly annotated to three phyla: *Ascomycota*, *Basidiomycota*, and *Zygomycota* [25,26,27]. Consistent with previous studies, our findings show two dominant phyla, *Ascomycota* and *Basidiomycota*, which have been reported to colonize the human GI tract, skin, vagina, and oral cavity [20,24]. The overall fungal community is lower than that of bacteria in the healthy human gut, and one study identified 66 fungal genera in fecal samples from 98 healthy individuals [26]. The present study identified 31 core fungal taxa, eight of which are the commonly reported gut fungi in gut mycobiome studies, including *Saccharomyces*, *Candida*, *Aspergillus*, *Malassezia*, *Penicillium*, *Cladosporium, Trichosporon*, and *Debaryomyces*, ranked in decreasing prevalence [28], although some of the fungal taxa might be transient members originating from the environment and diet. A recent study argued that fungal microbes found in stool samples could be attributed to dietary or oral sources [14]. Further investigation is necessary to investigate the growth potential of different fungal species in the intestine and their possible links with oral and dietary fungal communities.

Evidence from recent cohort studies and trials suggests that gut fungal composition can be directly driven by diverse dietary intake and patterns [11,26,29]. In an intervention study of 10 healthy individuals, the short-term plant-based diet altered the microbial community structure and was associated with enrichment in fecal *Candida* abundance [11]. Another cohort study of 98 individuals found that the abundance of *Candida* was strongly correlated with carbohydrate intake [26]. In mice, a high-fat diet could shift the gut fungal community structures with a lower abundance of the genus *Saccharomyces* but no change in *Candida* compared to standard chow [30]. We profiled the dynamics of fungal genera, such as *Saccharomyces*, *Aspergillus*, *Candida*, *Malassezia, Cladosporium,* and *Penicillium*, suggesting that the relative abundance of fungal genera exhibited substantial variation across participants in response to identical controlled diets.

The aggregated results from sequential n-of-1 trials allow us to distinguish gut mycobiome features responding to a specific intervention at a population level. Given that the washout periods returned the participants to the baseline state unaffected by the preceding treatment, the averaged relative abundance of core diet-related fungi exhibited diverse responses to the HC and LC dietary interventions. The present study identified enrichment of the fungal genus *Pleurotus*, *Auricularia*, and *Kazachstania* after an HC diet. Notable, we cannot rule out that some of the fungi may be transient members introduced from the foods (e.g., edible mushrooms and fermenting yeasts) rather than resident commensals truly colonizing in the human gastrointestinal tract. For example, the species of the genus *Pleurotus*, especially oyster mushrooms (*Pleurotus ostreatus)*, are among the most widely consumed edible mushrooms and possess hypoglycemic, antitumor, and antifungal activity [31,32]. The *Auricularia* genus and its well-known species, *Auricularia auricula*, have been reported as healthy edible fungi rich in bioactive compounds [33]. Moreover, *Kazachstania* is a common yeast genus capable of fermenting sugar and producing secondary metabolites, which are reported to be enriched in bakery sourdoughs [34,35]. Our study identified the genus *Penicillium* decreased considerably after an LC diet, which is consistent with a two-arm feeding study which reported a decrease in gut *Penicillium* after fat-rich pistachio consumption [13]. In addition, another study revealed a considerable enrichment of *Penicillium* and *Malassezia* among vegetarians compared to participants with a conventional Western diet [36]. Together, this evidence highlights the short-term changes in gut mycobiome are probably strongly influenced by the macronutrient distributions of diet. Nevertheless, large-scale studies in humans and the detailed mechanisms of the effect of macronutrients on the gut mycobiome, like *Penicillium*, with related health effects, are yet to be revealed in future.

Previous studies have examined gut mycobiome composition and its role in health and diseases in small trials or in cohorts of healthy individuals [14,24,37,38,39]. Certain fungal strains may be able to alter host metabolism, thereby influencing the pathogenesis and progression of diseases. Mycobiome dysbiosis has been reported in inflammatory diseases such as IBD and cardiometabolic diseases [7,27,40,41,42]. Yeast from the *Ustilaginaceae* family produce a range of value-added bio-products like carbohydrate-active enzymes (CAZymes) that can act on carbohydrates [43,44,45,46]. Furthermore, dietary changes can alter both the microbial composition and the associated CAZymes abundance [47]. Related to our findings, *Ustilaginaceae* sp. Carrying CAZymes that ferment carbohydrates may partially explain the association of *Ustilaginaceae* sp. Abundance with upregulated MPG and AUC_24_ under a high carbohydrate diet environment. Nevertheless, the detailed mechanism of the interaction of gut mycobiome with host uptake of macronutrient composition warrants further investigation.

Our present study has several strengths. First, the repeated cross-over design enables simultaneous comparisons between and within individuals to examine gut mycobiome variation and stability over time. Second, the nature of this feeding trial could help establish the causal effect of dietary intervention on the gut mycobiome. Third, the identified core fungi offer insights into the mechanisms underlying host-mycobiome interaction. Nevertheless, some limitations and unanswered questions remain. First, the long-term effect of dietary macronutrients on the gut mycobiome is still unclear, which could not be addressed in the present study. Second, the health effect of the identified dietary macronutrient-related gut fungi is still uncertain, and larger studies are needed to confirm their relationship with metabolic health. Moreover, the assessment of the abundance of fungal species in the gut based on fecal samples is not rigorously representative of the gut mycobiome, as we cannot rule out that some of the identified fungi may be transient members originating from the diet. Therefore, caution should be taken when interpreting the link between gut fungi and host health, especially when the identified fungi are less likely inhabiting in the digestive tract.

## 5. Conclusions

In summary, our present feeding-based clinical trial provides evidence of how short-term HC and LC dietary interventions affect gut mycobiome diversity and composition. Our results also indicate that the dietary environment may also influence the relationship between gut mycobiome and glycemic phenotypes, which warrants further confirmation in future.

## Figures and Tables

**Table 1:** Baseline characteristics of the WE-MACNUTR study participants.

	Overall (n = 28)
Age (years)	25.8 ± 2.7
BMI (kg/m^2^)	22.1 ± 3.0
Waist circumference (cm)	78.9 ± 9.1
Current drinking (%)	
Occasionally	6 (67)
Never	3 (33)
Fasting clinical parameters	
Insulin (mU/L)	25.4 ± 5.3
Fasting plasma glucose (mmol/L)	4.20 ± 0.26
Triglycerides (mmol/L)	0.77 ± 0.38
Total cholesterol (mmol/L)	4.30 ± 0.77
LDL cholesterol (mmol/L)	1.92 ± 0.59
HDL cholesterol (mmol/L)	1.67 ± 0.32

Data are presented as mean ± standard deviation or number (%). BMI, body mass index; LDL, low-density lipoprotein; HDL, high-density lipoprotein.

## Data Availability

The data used for analysis in this study are available from the corresponding author on request.

## Supplementary Materials

The following supporting information can be downloaded at: https://www.mdpi.com/article/10.3390/nu15092152/s1, Table S1: Taxonomic annotation results for gut mycobiome in the WE-MACNUTR study; Table S2: Fungal phylum with different relative abundance between trial arms; Table S3: Fungal genera with different relative abundance between trial arms; Table S4: Prevalence of core gut mycobiome detected at all sampling points; Table S5: PERMANOVA analysis results based on Bray–Curtis dissimilarity; Table S6: Short-term variation of the human gut mycobiome induced by the HC diet; Table S7: Short-term variation of the human gut mycobiome induced by the LC diet; Table S8: Associations between the mean relative fungal abundance and the mean values of glycemic responses in the HC diet (n = 28); Table S9: Associations between the mean relative fungal abundance and the mean values of glycemic responses in the LC diet (N = 28).
