# Supplementary material for: The Short-Term Variation of Human Gut Mycobiome in Response to Dietary Intervention of Different Macronutrient Distributions
Source: Nutrients. 2023 Apr 29;15(9):2152. doi: 10.3390/nu15092152 (PMC10180985; doi:10.3390/nu15092152)
Supplement: Supplementary file 1 [file nutrients-15-02152-s001.zip › Tian et al_CONSORT.pdf]

CENT 2015 checklist*; CONSORT 2010 checklist items with modifications or additions for individual or series of N-of-1 trials

Section/Topic	Item No	Checklist item	Reported on page No
**Title and abstract**			
	1a	Identify as an "N-of-1 trial" in the title<br>***For series:*** Identify as "a series of N-of-1 trials" in the title	1
	1b	Structured summary of trial design, methods, results, and conclusions (for specific guidance see CONSORT for abstracts)	1
**Introduction**			
Background and objectives	2a.1	Scientific background and explanation of rationale	1, 2
	2a.2	Rationale for using N-of-1 approach	1, 2
	2b	Specific objectives or hypotheses	2
**Methods**			
Trial design	3a	Describe trial design, planned number of periods, and duration of each period (including run-in and wash out, if applicable)<br>***In addition for series:*** Whether and how the design was individualized to each participant, and explain the series design	2
	3b	Important changes to methods after trial commencement (such as eligibility criteria), with reasons	2
Participants	4a	Diagnosis or disorder, diagnostic criteria, comorbid conditions, and concurrent therapies.<br>***For series:*** Same as CONSORT item 4a	2
	4b	Settings and locations where the data were collected	2
	4c	Whether the trial(s) represents a research study and if so, whether institutional ethics approval was obtained	12
Interventions	5	The interventions for each period with sufficient details to allow replication, including how and when they were actually administered	2
Outcomes	6a.1	Completely defined pre-specified primary and secondary outcome measures, including how and when they were assessed	2–4
	6a.2	Description and measurement properties (validity and reliability) of outcome assessment tools	2-4
	6b	Any changes to trial outcomes after the trial commenced, with reasons	Not applicable
Sample size	7a	How sample size was determined	2, Reference 12
	7b	When applicable, explanation of any interim analyses and stopping guidelines	Not applicable
Randomisation:			
Sequence generation	8a	Whether the order of treatment periods was randomised, with rationale, and method used to generate allocation sequence	2, Reference 12
	8b	When applicable, type of randomisation; details of any restrictions (such as pairs, blocking)	2, Reference 12

	8c	Full, intended sequence of periods	2, Reference 12
Allocation concealment mechanism	9	Mechanism used to implement the random allocation sequence (such as sequentially numbered containers), describing any steps taken to conceal the sequence until interventions were assigned	2, Reference 12
Implementation	10	Who generated the random allocation sequence, who enrolled participants, and who assigned participants to interventions	2, Reference 12
Blinding	11a	If done, who was blinded after assignment to interventions (for example, participants, care providers, those assessing outcomes) and how	2, Reference 12
	11b	If relevant, description of the similarity of interventions	Not relevant
Statistical methods	12a	Methods used to summarize data and compare interventions for primary and secondary outcomes	4
	12b	***For series:*** If done, methods of quantitative synthesis of individual trial data, including subgroup analyses, adjusted analyses, and how heterogeneity between participants was assessed, (for specific guidance on reporting syntheses of multiple trials, please consult the PRISMA Statement)	4
	12c	Statistical methods used to account for carryover effect, period effects, and intra-subject correlation	Not applicable
**Results**			
Participant flow (a diagram is strongly recommended)	13a.1	Number and sequence of periods completed, and any changes from original plan with reasons	4
	13a.2	***For series:*** The number of participants who were enrolled, assigned to interventions, and analysed for the primary outcome	4
	13b	***For series:*** losses or exclusions of participants after treatment assignment, with reasons, and period in which this occurred, if applicable	2, Reference 12
Recruitment	14a	Dates defining the periods of recruitment and follow-up	2, Reference 12
	14b	Whether any periods were stopped early and/or whether trial was stopped early, with reason(s).	Not applicable
Baseline data	15	A table showing baseline demographic and clinical characteristics for each group	4
Numbers analysed	16	For each intervention, number of periods analysed.<br>***In addition for series:*** if quantitative synthesis was performed, number of trials for which data were synthesized	3
Outcomes and estimation	17a.1	For each primary and secondary outcome, results for each period; an accompanying figure displaying the trial data is recommended.	5-9
	17a.2	For each primary and secondary outcome, the estimated effect size and its precision (such as 95% confidence interval)<br>***In addition for series:*** if quantitative synthesis was performed, group estimates of effect and precision for each primary and secondary outcome	5-9
	17b	For binary outcomes, presentation of both absolute and relative effect sizes is recommended	Not binary outcomes
Ancillary analyses	18	Results of any other analyses performed, including assessment of carryover effects, period effects, intra-subject correlation<br>***In addition for series:*** If done, results of subgroup or sensitivity analyses	2, Reference 12

Harms	19	All harms or unintended effects for each intervention. *(for specific guidance see CONSORT for harms)*	Not applicable
**Discussion**			
Limitations	20	Trial limitations, addressing sources of potential bias, imprecision, and, if relevant, multiplicity of analyses	11
Generalisability	21	Generalisability (external validity, applicability) of the trial findings	10, 11
Interpretation	22	Interpretation consistent with results, balancing benefits and harms, and considering other relevant evidence	10, 11
**Other information**			
Registration	23	Registration number and name of trial registry	11
Protocol	24	Where the full trial protocol can be accessed, if available	2, Reference 12
Funding	25	Sources of funding and other support (such as supply of drugs), role of funders	11

*We strongly recommend reading this statement in conjunction with the CONSORT 2010 Explanation and Elaboration for important clarifications on all the items. If relevant, we also recommend reading CONSORT extensions for cluster randomised trials, non-inferiority and equivalence trials, non-pharmacological treatments, herbal interventions, and pragmatic trials. Additional extensions are forthcoming: for those and for up to date references relevant to this checklist, see www.consort-statement.org.
